# Patient leaflets on respiratory tract infections did not improve shared decision making and antibiotic prescriptions in a low-prescriber setting

**DOI:** 10.1038/s41598-024-55166-7

**Published:** 2024-02-29

**Authors:** Andreas Plate, Stefania Di Gangi, Christian Garzoni, Kevin Selby, Giuseppe Pichierri, Oliver Senn, Stefan Neuner-Jehle

**Affiliations:** 1 Institute of Primary Care, University of Zurich and University Hospital Zürich, Pestalozzistrasse 24, 8091 Zurich, Switzerland; 2 mediX Ticino and Clinic of Internal Medicine and Infectious Diseases, Clinica Luganese Moncucco, Lugano, Switzerland; 3 Center for Primary Care and Public Health (Unisanté), Lausanne, Switzerland

**Keywords:** Public health, Respiratory tract diseases

## Abstract

Patient information leaflets can reduce antibiotic prescription rates by improving knowledge and encouraging shared decision making (SDM) in patients with respiratory tract infections (RTI). The effect of these interventions in antibiotic low-prescriber settings is unknown. We conducted a pragmatic pre-/post interventional study between October 2022 and March 2023 in Swiss outpatient care. The intervention was the provision of patient leaflets informing about RTIs and antibiotics use. Main outcomes were the extent of SDM, antibiotic prescription rates, and patients’ awareness/knowledge about antibiotic use in RTIs. 408 patients participated in the pre-intervention period, and 315 patients in the post- intervention period. There was no difference in the extent of SDM (mean score (range 0–100): 65.86 vs. 64.65, *p* = 0.565), nor in antibiotic prescription rates (no prescription: 89.8% vs. 87.2%, *p* = 0.465) between the periods. Overall awareness/knowledge among patients with RTI was high and leaflets showed only a small effect on overall awareness/knowledge. In conclusion, in an antibiotic low-prescriber setting, patient information leaflets may improve knowledge, but may not affect treatment decisions nor antibiotic prescription rates for RTIs.

## Introduction

Antimicrobial resistance (AMR) is a major public health threat^1^ and the inappropriate use of antibiotics is a main driver for rising AMR rates^2^. The vast majority of antibiotics is prescribed in the outpatient setting and respiratory tract infections (RTI) are the most common reason for prescribing antibiotics^3,4^. Of particular concern is the high degree of inappropriate antibiotic prescriptions in patients with RTI^5^. Accordingly, many interventions aim to improve the quality of care for patients with RTI in the outpatient setting^6,7^. One approach to improve the quality of care is to foster Shared Decision Making (SDM) during the medical consultation as SDM can reduce the amount of antibiotics prescribed in patients with RTI^6,8^. SDM is a process, in which both the patient and the health care provider (HCP) make a joint decision on further management. The decision is based on the available evidence and takes the patient values and preferences into account. A pre-requisite for SDM is the knowledge about facts, for example about benefits and disadvantages of several options, among patients and HCP^9^.

The Choosing Wisely® campaigns are well-known initiatives to promote quality of care in the medical field according to the principle of “less is more”^10^. One of the most prominent recommendations in the field of general internal medicine is to avoid antibiotics for uncomplicated upper respiratory tract infections. In Switzerland, the campaign provides a patient information leaflet on this recommendation for the use in the primary care setting^11^. Patient information leaflets are well known tools to reduce antibiotic consumption in primary care^12^. The leaflet on RTI contains written text and graphic elements and provides basic knowledge for patients about the etiology of RTI. It explains in plain language why antibiotics are considered inappropriate for the treatment of RTI, in order to make patients aware that the avoidance of an antibiotic treatment may be appropriate in their current condition. Thus, the leaflets are intended to serve as a support tool or decision aid for both patients and HCP fostering SDM.

In Europe, Switzerland is one of the countries with the lowest antibiotic consumption rates^13^. The effect of such leaflets in an antibiotic low-prescriber setting is unknown. Thus, the aim of this study was to evaluate the effect of the leaflet in a low-prescriber setting, among patients with RTIs. We aimed to determine the effect of the leaflet on the extent of SDM and on antibiotic prescribing rates. In addition, we aimed to evaluate its effect on the knowledge and awareness of antibiotic prescribing inappropriateness as well as the perception of the leaflets among patients.

## Methods

### Study design and setting

We conducted a pragmatic pre-post intervention study from October 2022 to March 2023: pre- intervention period (October–December 2022) and post- intervention period (January–March 2023). The Swiss flu season typically peaks between the end of January and beginning of March. In the 2022–2023 season the peak was in late December^14^.

### Study participants and eligibility criteria

Physicians providing primary care and affiliated with the local study centers in one of the three language regions of Switzerland were invited by e-mail to participate in the study. The invitations provided information on the study aims and procedures. Study physicians already using the Choosing Wisely® leaflets or other leaflets / decision aids for antibiotic treatment decisions during consultations on a regular basis were excluded. Patients of participating physicians with one of the following RTIs were eligible for participation: rhinitis, sinusitis, pharyngitis, tonsillitis, bronchitis, influenza, streptococcal pharyngitis and Covid-19. A positive rapid test was a mandatory condition for the inclusion of patients with the diagnoses of influenza, streptococcal pharyngitis or Covid-19. The remaining diagnoses could be made based on patient history and clinical examination. Patients could participate in the study more than once, if they had more encounters.

### Data sources and measurements

Data was collected from both patients and physicians. Patient data were collected via a self-administered, open online questionnaire developed in English and then translated into three languages: French, German, Italian, according to the main language spoken in the residence area of recruiting study physicians. RedCap study software^15,16^ was used to host and manage the survey. At the end of the consultation with their GP, eligible patients received an invitation letter to participate in the study. The invitation letter contained information on the background and aims of the study as well as a QR code and web link to the online questionnaire. Alternatively, patients had the opportunity to complete a pen-and-paper version of the survey. Pen-and-paper questionnaires were sent to the study center and data were transcribed into the study software. An independent study staff member reviewed all data entry. The questionnaire contained questions on four topics: 1) reason for the current consultation, duration of symptoms and questions about antibiotic treatments 2) awareness/knowledge about antibiotic use and AMR, 3) extent of SDM during the medical consultation, and 4) patient characteristics and medical history, i.e. comorbidities. Questionnaires in the post-intervention period contained in addition questions about the leaflets. The section on AMR included eleven statements regarding definition and awareness/knowledge. Statements were derived from questionnaires on AMR^17,18^. A English translation of the survey as well as the Checklist for Reporting Results of Internet E-Surveys (CHERRIES Checklist) is provided in the supplemental^19^. Study physician characteristics were collected through a self-administered online survey. After patient recruitment was completed, all study physicians were invited to participate in the post-study evaluation. Data were also collected via a self-administered, online questionnaire.

### Intervention

The intervention consisted of passive exposure of patients to the leaflets and in addition basic information about SDM for physicians. The leaflets were placed in the patient waiting rooms. Additional leaflets could be placed at the reception desk, in examination rooms, in the doctor’s room, or digitally on screens (as screen savers) in the practice. In addition, practices were offered the leaflets in poster format. All study physicians received a laminated version of the leaflet as a visual aid for use during the consultation. The use of the laminated version was voluntary. All study physicians were provided with written information about the leaflets at the beginning of the intervention-period. It contained information on the content and key messages of the leaflets as well as the information that the intention of the leaflets is to foster the process of SDM. In addition, all study physicians were provided with basic information about SDM and how to use during the consultation in general.

### Outcomes

The primary outcome was the extent of SDM during the medical consultation in the pre- and post- intervention period. The validated 9-item Shared Decision Making Questionnaire (SDM-Q-9)^20^ was used to measure the extent of SDM. Each item, featuring an aspect of SDM, rated on a 6-point balanced scale ranging from 0 (= ‘completely disagree’) to 5 (= ‘completely agree’) with the possibility to select “no answer”. The total score, sum of the score of the nine items, ranged between 0 and 45 and, according to the literature^20^, was rescaled to a 0–100 range. As no generally accepted standard of good SDM exists and no comparative literature is available for this particular setting, no effect size for the primary outcome was assumed (and therefore no sample size was calculated).

Secondary outcomes were the proportion of antibiotics prescribed, patient knowledge and awareness of AMR, patient perception rate of the leaflets, and patient and study physician experiences with the leaflets. Patient knowledge and awareness of AMR were evaluated through statements on the inappropriateness of antibiotic prescribing and notion of antibiotic resistance. For each item we provided a Likert scale from 1 (= ’completely disagree’) to 5 (= ‘completely agree’) with the possibility to select “no answer”. Subgroup of patients in the post-intervention were identified in dependence on whether they reported to have seen the leaflets (yes/no/don’t remember). Study physician and patient experiences were evaluated through statements in Likert scale from 1 (= ‘completely disagree’) to 5 (= ‘completely agree’) with the possibility to select “no answer”.

### Statistical methods

Descriptive statistics were presented as means (standard deviations [SD]) for continuous variables and as number N(%), for categorical variables. Differences between pre and post intervention groups were tested using t-test for continuous variables and chi-square or Fisher exact test, as appropriate, for categorical variables. Variables defined as Likert-scale points were visualized though a stacked centered bar chart, with the total % of disagreement (points 1 and 2), neutrality (point 3 and no answer) and agreement (points 4 and 5). Subgroups differences (pre-post intervention and pre-post intervention with or without leaflets) in disagreement/agreement rates were tested using chi-square or Fisher exact test. All statistical analyses were carried out using statistical package R, R Core Team (2016), version 4.1.0.^21^. In particular we used Likert package for visualization.

Missing data: All available data were analyzed and the number of missing data were reported when necessary. The category “no answer”, where stated, was not considered as missing information. For calculation of the SDM-Q-9 total score up to two missing items values were imputed to calculate the raw score. Imputed values were the mean of the available results. SDM-Q-9 data with three or more missing entries were excluded from analysis^20^.

Patients who reported previous participation in the study were excluded from the analyses of knowledge and awareness of AMR and SDM items in order to avoid confounding by unintended learning effects. Patients (*n* = 5) of two study physicians were excluded from post-intervention analysis, as study physicians accidentally placed the leaflets only in other practice rooms, but not in the waiting room itself.

All the methods were performed in accordance with the relevant institutional guidelines and regulations.

### Ethics approval and Consent to participate

The study did not fall under the scope of the national human research act. Thus, the need for ethics approval was exempted by the competent ethics committee of Zurich, Switzerland (BASEC number: Req-2022-00,369). Participation in the survey was voluntary. On the first page of the questionnaire, all patients were informed about the purpose of the study and the anonymity in case of participation. Patients gave their informed consent before access to the questionnaire was given.

## Results

### Study physician and patient characteristics

A total of *n* = 57 study GPs recruited a total of *n* = 723 patients (56.4% female, mean age 47.1 (SD: 17.7) years). Most common patient diagnosis were Rhinitis / Rhinosinusitis / Sinusitis (*n* = 299 patients, 41.5%), and Bronchitis (*n* = 284 patients, 39.3%). Basic study physician and patient characteristics are presented in Table 1.

**Table 1:** Basic characteristics of study physicians and patients (Leaflets for respiratory tract infections, Switzerland, 2022–2023).

Study physician characteristics, n = 57*				
Female gender		21 (36.8)		
Age (years)	Mean (SD)	47.51 (10.34)		
Work experience (years)	Mean (SD)	14.65 (9.53)		
Work load (%)	Mean (SD)	81.58 (17.73)		
Type of practice				
Single		13 (22.8)		
Double		6 (10.5)		
Group		33 (57.9)		
Walk in		5 (8.8)		
Affiliated to a medical network		49 (86.0)		

Patient characteristics		Pre- Intervention period	Post- intervention period	*p*
Patients recruited		408	315	
Sex				0.307
Female		212 (54.9)	173 (58.2)	
Male		170 (44.0)	124 (41.8)	
Other		3 (0.8)	0 (0.0)	
Prefer not to say		1 (0.3)	0 (0.0)	
Age (years)	Mean (SD)	47.85 (18.19)	46.14 (16.90)	0.21
Language region				0.003
German		273 (66.9)	238 (75.6)	
French		33 (8.1)	31 (9.8)	
Italian		102 (25.0)	46 (14.6)	
Duration of symptoms before physician consultation (days)	Mean (SD)	6.28 (7.33)	6.80 (6.73)	
Time spent in the waiting room				0.144
< 15 min		289 (71.4)	199 (63.4)	
> 30 min		30 (7.4)	33 (10.5)	
15–30 min		84 (20.7)	80 (25.5)	
Don‘t remember		2 (0.5)	2 (0.6)	
Time from consultation to survey (days)				0.071
0		216 (53.1)	160 (51.0)	
1–5		146 (35.9)	101 (32.2)	
> 5		45 (11.1)	53 (16.9)	
Smoking status				0.092
Former smoker		130 (33.7)	91 (30.6)	
Never smoked		183 (47.4)	164 (55.2)	
Active smoker		73 (18.9)	42 (14.1)	
Comorbidities				
Lung disease		18 (4.4)	20 (6.3)	0.326
Cardiovascular disease		9 (2.2)	5 (1.6)	0.741
Diabetes mellitus		11 (2.7)	8 (2.5)	1
Cancer disease		6 (1.5)	4 (1.3)	1
Kidney disease		6 (1.5)	0 (0.0)	0.08
No chronic disease		325 (79.9) ara>	244 (77.5)	0.491
Diagnosis				
Rhinitis / Rhinosinusitis / Sinusitis		168 (41.3)	131 (41.9)	0.937
Pharyngitis / Tonsillitis		117 (28.7)	98 (31.1)	0.53
Bronchitis		146 (35.8)	138 (43.8)	0.035
Streptococcal pharyngitis		18 (4.4)	16 (5.1)	0.808
Influenza		22 (5.4)	26 (8.3)	0.167
Covid-19		54 (13.2)	11 (3.5)	< 0.001

*GPs recruited: 60. GPs drop-out’s: 3.

Data are presented as absolute numbers and percentage (in brackets) if not stated else.

Missing data (pre- / post- intervention period): Sex, Age, smoking status: 22/18; days from consultation to survey: 1/1; duration of symptoms, time spent in the waiting room: 3/1, Comorbidities: 1/0; Diagnosis 1/2.

8 patients (1.1%) reported to have participated more than once in the study.

### SDM-Q-9

The overall SDM-Q-9 score could be calculated in *n* = 323 (79.2%) and *n* = 242 (76.8%) patients of the pre- and post-intervention period, respectively. Compared to the pre-intervention period, we found no significant difference in the extent of SDM in the post- intervention period, neither in the overall group (65.86 vs. 64.65, *p* = 0.565) nor in the subgroup of patients that reported having seen the leaflets (65.86 vs. 66.07, *p* = 0.941) (Table 2 and supplemental Table 1).

**Table 2:** Results of the 9-item shared decision making questionnaire for patients (Leaflets for respiratory tract infections, Switzerland, 2022–2023).

Item raw scores (range 0–5)	Pre- intervention period	Post- intervention period			
		All patients	*p*	Leaflet subgroup*	*p*
My doctor made clear that a decision needs to be made	2.52 (1.85)	2.50 (1.73)	0.918	2.46 (1.80)	0.767
My doctor wanted to know exactly how I want to be involved in making the decision	2.85 (1.75)	2.72 (1.75)	0.388	2.62 (1.85)	0.249
My doctor told me that there are different options for treating my medical condition	3.10 (1.68)	3.15 (1.66)	0.712	3.20 (1.64)	0.565
My doctor precisely explained the advantages and disadvantages of the treatment options	3.34 (1.60)	3.34 (1.56)	0.997	3.50 (1.45)	0.312
My doctor helped me understand all the information	3.95 (1.40)	4.04 (1.25)	0.401	4.11 (1.17)	0.267
My doctor asked me which treatment option I prefer	3.17 (1.73)	2.95 (1.74)	0.132	3.12 (1.76)	0.791
My doctor and I thoroughly weighed the different treatment options	3.27 (1.59)	3.13 (1.61)	0.278	3.21 (1.60)	0.710
My doctor and I selected a treatment option together	3.34 (1.61)	3.37 (1.64)	0.811	3.38 (1.63)	0.822
My doctor and I reached an agreement on how to proceed	3.96 (1.36)	3.87 (1.36)	0.405	3.83 (1.47)	0.368
Total score (range 0–100)	65.86 (25.38)	64.65 (23.97)	0.565	66.07 (24.16)	0.941

Values are presented as mean (Standard deviation). *: Subgroup of patients who reported to have actively perceived the leaflets.

### Antibiotic prescriptions

In the pre-intervention period *n* = 362 (89.8%) patients reported not having received an antibiotic prescription compared to *n* = 273 (87.2%) in the post-intervention period (Table 3). We found no significant difference in the proportion of patients prescribed antibiotics for any specific diagnosis. With the exception of patients with streptococcal pharyngitis, antibiotics were prescribed in less than 12% of patients. The most common used antibiotic drugs were amoxicillin/ clavulanic acid (*n* = 35 (50.7%) prescriptions), and amoxicillin (*n* = 20 (29%) prescriptions) (Supplemental Table 2).

**Table 3:** Antibiotic prescription rates (Leaflets for respiratory tract infections, Switzerland, 2022–2023).

	Pre- intervention period	Post- intervention period: All patients		Post- intervention period: Leaflet subgroup*	
Overall prescription patterns					
No antibiotic prescription	362 (89.8)	273 (87.2)	0.465	117 (86.7)	0.268
Antibiotic prescription	34 (8.4)	35 (11.2)		17 (12.6)	
Don’t remember	7 (1.7)	5 (1.6)		1 (0.7)	
Proportion in patients with an antibiotic treatment stratified by diagnosis					
Rhinitis / Rhinosinusitis / Sinusitis	11 (6.5)	13 (10.0)	0.180	7 (11.7)	0.273
Pharyngitis / Tonsillitis	7 (6.0)	8 (8.2)	0.802	4 (9.8)	0.432
Bronchitis	6 (4.3)	10 (7.3)	0.552	5 (7.7)	0.477
Streptococcal pharyngitis	13 (72.2)	10 (62.5)	0.812	5 (71.4)	1.000
Influenza	1 (4.5)	0 (0.0)	0.146	0 (0.0)	0.146
COVID-19	2 (3.7)	0 (0.0)	0.648	0 (0.0)	0.648

Values are shown as absolute numbers and percentage. *: Subgroup of patients who reported to have actively perceived the leaflets. Missing data (pre- / post- intervention period): Prescription pattern (5/1).

### Knowledge and awareness

**Figure 1:**
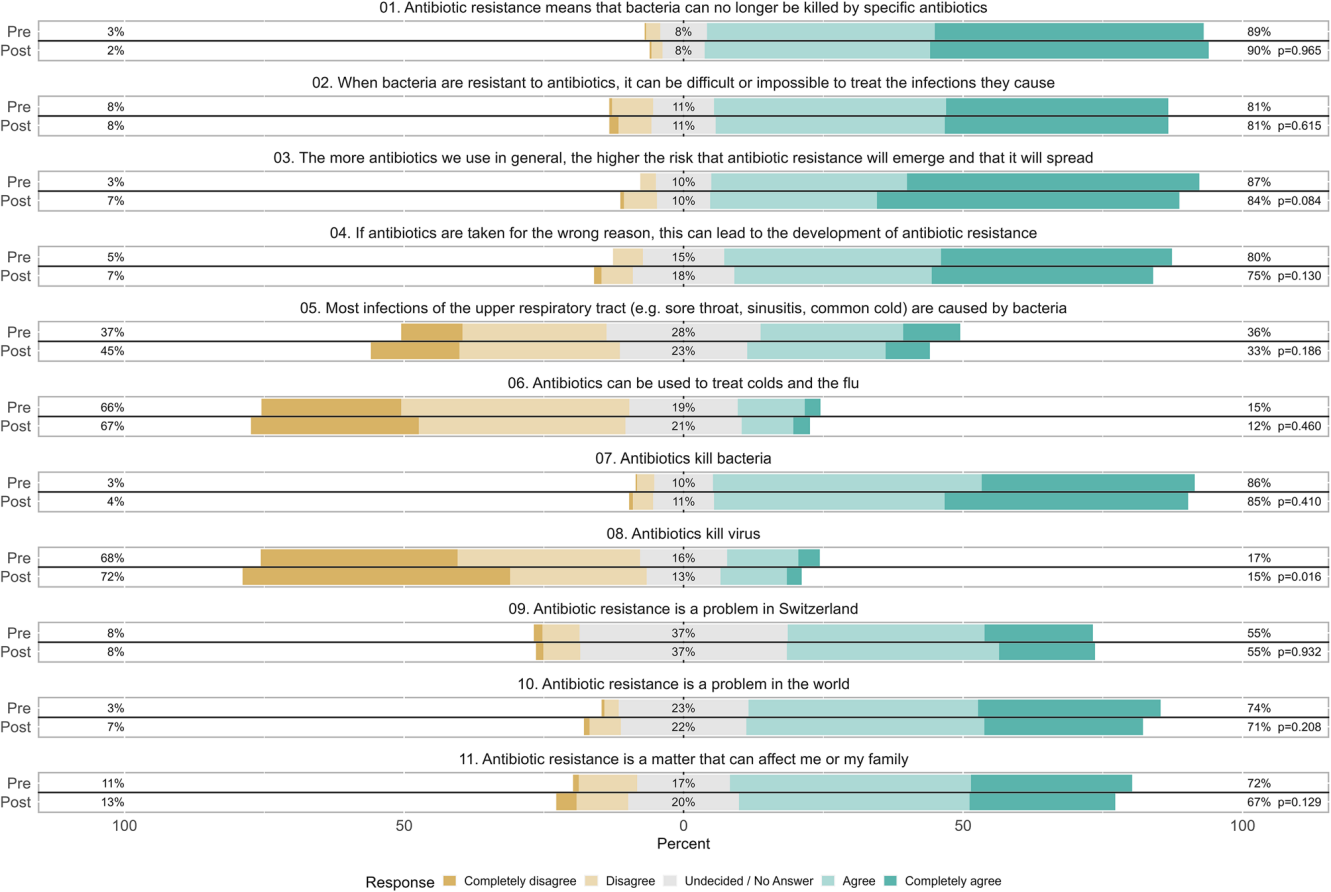
Patient ratings of knowledge and awareness statements. Pre: Patients in the pre-intervention period. Post: Patients in the post-intervention period. (Leaflets for respiratory tract infections, Switzerland, 2022–2023).

Results of patients rating of the knowledge and awareness statements are presented in Fig. 1. The statement “*antibiotics kill viruses*” was the only one with a significant improvement in terms of a higher proportion of respondents in the post- intervention period (compared to the pre-intervention period) rejecting the statement (*p* = 0.016). Results of subgroup analyses, stratified by respondents in the post-intervention period who reported to have seen the leaflets in the practice (yes/no/don’t remember) are shown in supplemental Fig. 1. In the subgroup that reported to have seen the leaflets, we found significant improved ratings to five statements (*antibiotic resistance means that bacteria can no longer be killed by specific antibiotics*, *p* = 0.041; *The more antibiotics we use in general, the higher the risk that antibiotic resistance will emerge and that it will spread*, *p* < 0.001; *Most infections of the upper respiratory tract (e.g. sore throat, sinusitis, common cold) are caused by bacteria*, *p* = 0.003; *antibiotics kill bacteria*, *p* = 0.033; *antibiotics kill viruses*, *p* < 0.001).

### Patient and study physician experiences with the leaflets

**Figure 2:**
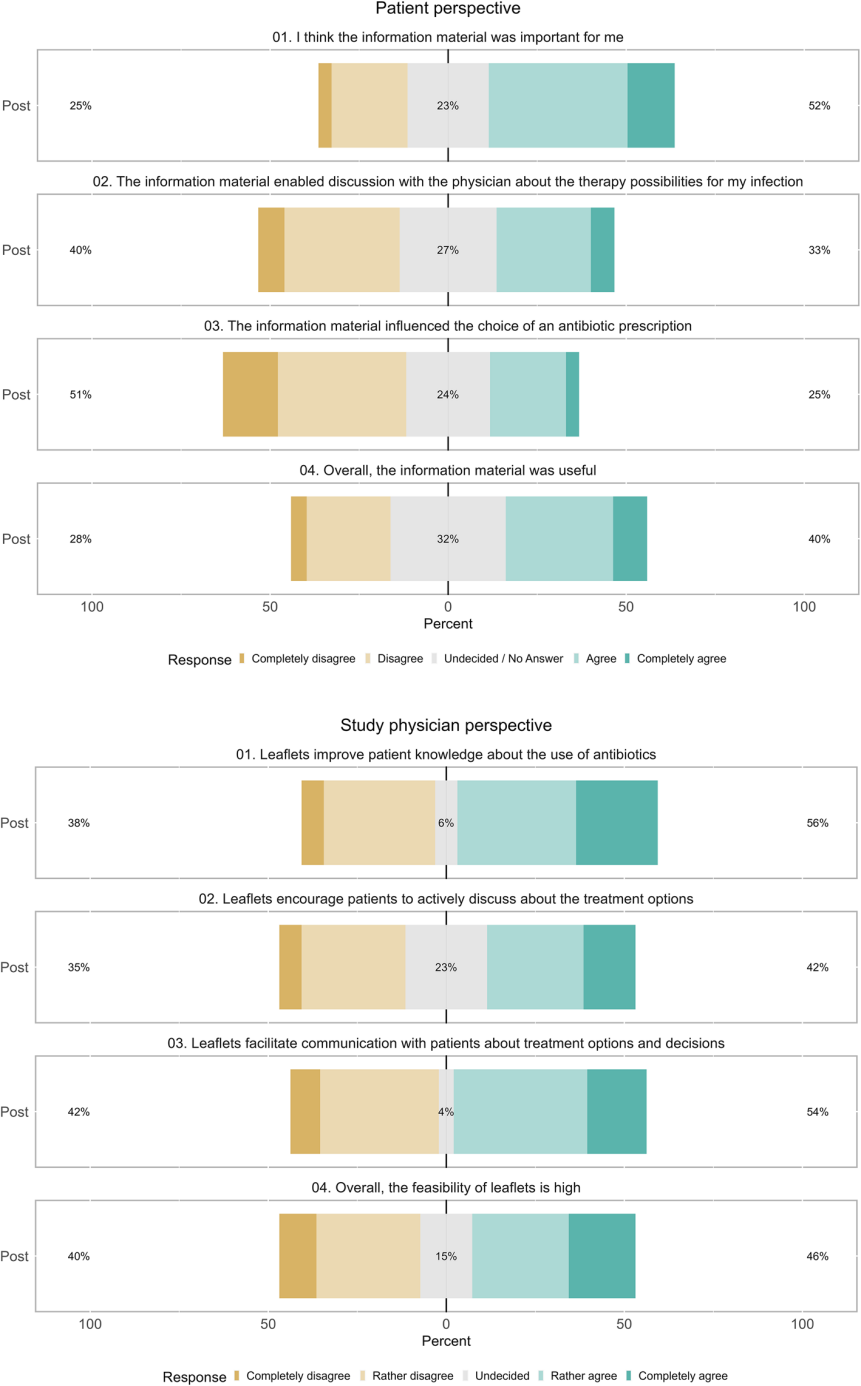
Patient and study physician statements on the leaflets (Leaflets for respiratory tract infections, Switzerland, 2022–2023).

In the post-intervention period *n* = 136 patients (44.3%) reported to have perceived the leaflets, while *n* = 97 patients (31.6%) did not notice the leaflets and *n* = 74 patients (24.1%) could not recall it. Patient statements of those patients who have seen the leaflets are shown in Fig. 2. Although a majority of patients reported that the leaflets were important (52% of all responses) and useful (40%), a majority also reported that the leaflets neither enabled discussions with their physicians (40%) nor influenced the choice of the therapy (51%). Free comments on the flyers were provided by *n* = 32 patients (23.5%). Three aspects were repeatedly mentioned: First, patients would like to see these leaflets more prominently displayed in practice and they would prefer to be made more aware of these leaflets by the practice staff. Second, patients recommended to place the leaflets not only in the waiting room as patients might bypass the waiting room during their appointment. Third, patients recommended providing these information’s in other locations, such as pharmacies, too.

Study physician participation rate in the post study evaluation survey was 84.2% (*n* = 48). Less than half of the study physicians (*n* = 19, 41%) reported to have at least once drawn the attention of patients to the leaflets. However, *n* = 37 (77%) mentioned that in the post-intervention period (compared to the pre-intervention period) patients did not wish for a discussion about possible treatment options more frequently. Study physicians’ rating of statements on the leaflets are shown in Fig. 2. Overall, there was a balanced ratio of study physicians who rated the leaflet and their effects as positive or as negative.

Study physicians reported having rarely (< 20%) used the laminated version of the leaflet as a visual tool during the consultation. Patients with bronchitis and rhinitis and / or sinusitis were the patient groups where study physicians saw the most benefit in using the leaflets. Overall usefulness of the leaflets was rated between moderate and rather high. However, the majority of study physicians will probably use the leaflets beyond the scope and duration of the study and will likely recommend the leaflets to other physicians (Supplemental Table 3).

## Discussion

In this pragmatic study, we determined the effects of a publicly available patient information leaflet on SDM, antibiotic prescription rates, and knowledge and awareness of AMR, among patients with RTIs, in an antibiotic low-prescriber setting. We found no effect, neither on the extent of SDM nor on antibiotic prescription rates, after passive exposure of patients to leaflets, and small effects on awareness/knowledge in patients having seen the leaflets.

Patient information materials, such as leaflets, are highly appreciated by patients^22^. Patients usually notice the leaflets in medical practices and report a positive effect on knowledge, self-management and patient–physician interaction^23^. Although there is good evidence that the use of patient leaflets and the promotion of SDM reduce antibiotic prescription rates in patients with RTI in primary care^6,7,12,24^ we found in our study neither a significant improvement in the extent of SDM nor in antibiotic prescription rates. We see two factors as decisive for this result: The clinical setting in which the study was conducted and the pragmatic study approach.

As seen during our pre-intervention period, the study took place in a clinical setting where SDM routinely is implemented to a high degree, where awareness/knowledge of patients about antibiotics and RTIs seems to be high, and where antibiotic prescription rates are low. Overall SDM-Q-9 scores in our study were similar to studies conducted in primary care settings^25–27^. One study on RTI reported higher SDM-Q-9 scores, but was focused on parents of children with RTI^28^. The majority of patients indicated that the leaflets, although important and useful, did not enable discussions about treatment options with physicians. This finding is in line with our observation that within the items of the SDM-Q-9 score, highest agreement was reached in two key statements, namely that physicians helped patients to understand all available information and that a consensus on how to proceed was reached. In addition, the missing effect on SDM could be caused by the fact, that a relevant proportion of patients in Swiss primary care with RTI do not prefer SDM during their consultation^29^. However, it is important to note that the Leaflets have not undergone formal user-testing or evaluation in clinical practice, which may also explain the observations.

Next, knowledge and awareness of AMR among study participants was already high. Across all statements, the vast majority of participants showed an adequate agreement / disagreement to the given statements. Finally, Switzerland has the second lowest outpatient antibiotic consumption rate in Europe^13^. Disease specific prescription rates in our study were far below the recommended thresholds for acute bronchitis (acceptable range: up to 30%), tonsillitis (20%), sinusitis (20%) or acute upper respiratory tract infections in general (20%) as defined by the European Surveillance of Antimicrobial Consumption^30^. Compared to the many other European countries disease specific antibiotic prescribing rates are low in our study^31,32^. Previous studies on SDM in primary care in patients with RTI were conducted in countries with higher antibiotic prescription rates^8^.

In addition to these effects, the lacking or small effects in our study are likely due to the pragmatic study design. Many studies have demonstrated effects of interventions based on increased SDM or provision of leaflets. However, these interventions were generally part of multifaceted interventions and the use and promotion of the leaflets were mandatory. The goal of this study was to assess the leaflets into routine clinical practice, as it would be feasible outside of thoroughly regulated studies. The limited time resources of physicians in routine care given, the nature of the exposure of leaflets to the patients was passive, and the use of the leaflets as an educational tool during the consultation was voluntary for the study physicians.

The process of a (shared) treatment decision is complex, consisting of many steps and depending on many factors, of which our intervention did address awareness and knowledge only. Correspondingly, the intention of our pragmatic study was not to evaluate a comprehensive decision aid covering all aspects of the SDM process, but to test out the “lower threshold of impact” of simple interventions guiding antibiotic treatment decisions. Specifically, to increase knowledge among patients, thereby triggering the process of SDM. If it were possible to show an effect for such minimal interventions, they may be an interesting alternative to more complex interventions in terms of feasibility, acceptance and the potential of their implementation in routine care.

### Implications for clinical care and future research

Almost half of all participants in the post-intervention period noticed the leaflets in the waiting rooms. In this subgroup, we observed increased knowledge and awareness of AMR. Our study results suggest a need for “enhanced exposure”. Accordingly, awareness could be increased by placing the leaflets not only in the waiting room, but systematically in areas where patients spend time. Depending on the organization of the practice, patients may not even need to go to the waiting area, but directly to the examination room or the doctor’s office. There is considerable variation in antibiotic prescribing frequencies among Swiss physicians^33^ and disease specific prescribing rates are known to be much higher in physicians with high volume of antibiotic prescriptions^34^. Future studies should evaluate whether passive intervention may have a clinical relevant effect in these specific settings, such as high-prescriber settings or in patients who have less knowledge about AMR. Furthermore, given the limited reach of passive exposure and patients’ desire for more active information, a more active use of leaflets could be more effective, but this must be balanced against the increased time required and therefore its acceptability by physicians.

The focus of the intervention could be adapted. In a context with a relatively low overuse of antibiotics, the expected effect may be rather small. Consequently, one should consider directing the focus of the intervention to conditions with higher antibiotic prescription rates (for example, streptococcal pharyngitis or acute otitis media). Finally, the leaflets should undergo a formal evaluation to improve their content if needed.

Two findings independently of the leaflets confirm the need for further antibiotic stewardship efforts, even in a low prescriber setting. First, amoxicillin in combination with clavulanic acid accounted for half of the prescriptions. The high proportion is well known for Swiss outpatient care^13,33,35^. Interventions should foster awareness of the appropriate use of amoxicillin without clavulanic acid in many clinical situations, as recommended in the respective national and international guidelines. Second, to the best of our knowledge, our study showed for the first time the proportion of patients with streptococcal pharyngitis in Switzerland who are treated without antibiotics. The antibiotic-free management of streptococcal pharyngitis is in line with national guidelines^36^ and it is encouraging to observe that almost one third of patients are managed without an antibiotic treatment during the initial consultation.

### Strengths and limitations of the study

A strength is the pragmatic approach of the study. The study was designed and conducted with the intention to test an intervention, which had great potential to be implemented later on in a real clinical setting. Second, the participating study physicians had similar characteristics to other general practitioners in Switzerland^37^. However, representativeness cannot be inferred. Third, since it is generally more difficult to achieve effects in low-prescriber settings, the results of the study are of great interest for various HCP and stakeholders to inform future interventions in similar settings. We see the greatest limitation due to the lower recruitment numbers in the post-intervention period. This could be explained by the fact that the peak of patients with influenza like illnesses in the 2022/2023 season was earlier than during the previous years, i.e. during the pre-intervention period. However, it is unlikely that increased recruitment numbers in the post-intervention period would have led to clinically relevant effects. Further, the results of the physician survey did not support physician drop-out due to low acceptability of the intervention. Finally, we have to acknowledge that some GPs may have been aware of the recommendation before the study, as the recommendation to withhold antibiotics for uncomplicated RTI has already been published by the national Choosing Wisely® campaign in 2014 and 2020.

## Conclusion

Passive exposure of patients with information leaflets is a minimal and feasible intervention. In an antibiotic low-prescriber setting, patient information leaflets may improve knowledge, but may not impact on treatment decisions nor antibiotic prescription rates for RTIs. However, half of the study patients noticed the leaflets and they acknowledged their usefulness and the importance of the AMR topic. It should be further investigated whether information leaflets which have been evaluated by the target groups and which are focused on specific settings, such as high-prescriber practices, could result in clinically relevant effects. Furthermore, it is important to determine whether actively exposing patients to the leaflets by physicians is well-received and thus feasible and if it leads to a clinically significant impact.

### Supplementary Information

Supplementary Information.

## Data Availability

The datasets used and/or analyzed during the current study available from the corresponding author on reasonable request.
