# Supplementary material for: Patient leaflets on respiratory tract infections did not improve shared decision making and antibiotic prescriptions in a low-prescriber setting
Source: Sci Rep. 2024 Feb 29;14:4978. doi: 10.1038/s41598-024-55166-7 (PMC10904372; doi:10.1038/s41598-024-55166-7)
Supplement: Supplementary file 1 — Supplementary Information. [file 41598_2024_55166_MOESM1_ESM.docx]

**Online Supplemental**

Title: Patient leaflets on respiratory tract infections did not improve shared decision making and antibiotic prescriptions in a low-prescriber setting.

| **Supplemental table 1: Number of available answers to calculate the SDM-Q-9** (Leaflets for respiratory tract infections, Switzerland, 2022-2023) | | | |
| --- | --- | --- | --- |
| Item | Pre- intervention period | Post- intervention period: all patients | Post- intervention period: Leaflet subgroup* |
| My doctor made clear that a decision needs to be made. | 320 | 248 | 114 |
| My doctor wanted to know exactly how I want to be involved in making the decision. | 316 | 242 | 111 |
| My doctor told me that there are different options for treating my medical condition. | 346 | 273 | 122 |
| My doctor precisely explained the advantages and disadvantages of the treatment options. | 331 | 268 | 119 |
| My doctor helped me understand all the information | 361 | 272 | 121 |
| My doctor asked me which treatment option I prefer. | 343 | 258 | 117 |
| My doctor and I thoroughly weighed the different treatment options. | 325 | 255 | 116 |
| My doctor and I selected a treatment option together. | 339 | 269 | 120 |
| My doctor and I reached an agreement on how to proceed. | 348 | 273 | 122 |
| **Number of participants included for total score calculation** | 323 | 242 | 109 |
| *: Subgroup of patients who reported to have actively perceived the leaflets. | | | |

| **Supplemental table 2: Overview of prescribed antibiotic drugs (% over the patients with prescriptions)** (Leaflets for respiratory tract infections, Switzerland, 2022-2023) | | | |
| --- | --- | --- | --- |
| Antibiotic drug | Pre- intervention period | Post- intervention period | |
|  |  | All patients | Leaflet subgroup* |
| Amoxicillin | 10 (29.4) | 10 (28.6) | 6 (35.3) |
| Amoxicillin / Clavulanic acid | 17 (50.0) | 18 (51.4) | 8 (47.1) |
| Azithromycin ** | 2 (5.9) | 3 (8.6) | 1 (5.9) |
| Moxifloxacin ** | 1 (2.9) | 0 (0.0) | 0 (0.0) |
| Trimethoprim / Sulfamethoxazole | 0 (0.0) | 1 (2.9) | 1 (5.9) |
| Cefuroxime | 1 (2.9) | 1 (2.9) | 1 (5.9) |
| Clarithromycin | 2 (5.9) | 2 (5.7) | 0 (0.0) |
| Phenoxymethylpenicillin | 2 (5.9) | 0 (0.0) | 0 (0.0) |
| Values are presented as absolute numbers and percentage. *: Subgroup of patients who reported to have actively perceived the leaflets. **: One patient reported to have received Azithromycin in combination with Moxifloxacin | | | |
|  | | | |

| **Supplemental Table 3: Study physician. Post study evaluation** (Leaflets for respiratory tract infections, Switzerland, 2022-2023) | | | | | | |
| --- | --- | --- | --- | --- | --- | --- |
| Use of the leaflets in patient groups | Patient groups | | | | | |
|  | Rhinitis / Rhinosinusitis / Sinusitis | Pharyngitis / Tonsillitis | Bronchitis | Streptococcal pharyngitis | Influenza | Covid-19 |
| <20% | 29 (60.4) | 29 (60.4) | 28 (58.3) | 33 (68.8) | 29 (60.4) | 33 (68.8) |
| 21-40% | 10 (20.8) | 7 (14.6) | 9 (18.8) | 6 (12.5) | 5 (10.4) | 3 (6.2) |
| 41-60% | 5 (10.4) | 5 (10.4) | 3 (6.2) | 2 (4.2) | 4 (8.3) | 1 (2.1) |
| 61-80% | 1 (2.1) | 5 (10.4) | 5 (10.4) | 3 (6.2) | 3 (6.2) | 4 (8.3) |
| >80% | 2 (4.2) | 1 (2.1) | 1 (2.1) | 2 (4.2) | 2 (4.2) | 2 (4.2) |
| No patients with the diagnosis | 0 (0.0) | 0 (0.0) | 0 (0.0) | 1 (2.1) | 3 (6.2) | 3 (6.2) |
| Don't know | 1 (2.1) | 1 (2.1) | 2 (4.2) | 1 (2.1) | 2 (4.2) | 2 (4.2) |
|  | | | | | | |
| Patients groups for which physicians see the most benefit when using the leaflets | 21 (43.8) | 19 (39.6) | 28 (58.3) | 5 (10.4) | 18 (37.5) | 11 (22.9) |
|  | | | | | | |
| Overall usefulness of the leaflets | | | | | | |
| Very high | 3 (6.2) | | | | | |
| Rather high | 15 (31.2) | | | | | |
| Middle | 19 (39.6) | | | | | |
| Rather low | 7 (14.6) | | | | | |
| Low | 4 (8.3) | | | | | |
| Using the leaflet beyond the purpose of the study | | | | | | |
| Very likely | 6 (12.5) | | | | | |
| Likely | 24 (50.0) | | | | | |
| Undecided | 7 (14.6) | | | | | |
| Rather not likely | 4 (8.3) | | | | | |
| Very unlikely | 7 (14.6) | | | | | |
| Would physicians recommend the use of the leaflet to other physicians? | | | | | | |
| Very likely | 3 (6.2) | | | | | |
| Likely | 22 (45.8) | | | | | |
| Undecided | 9 (18.8) | | | | | |
| Rather not likely | 9 (18.8) | | | | | |
| Very unlikely | 5 (10.4) | | | | | |

**Supplemental Figure 1:** Rating of knowledge and awareness statements. Pre: Patients in the pre-intervention period. Post: Patients in the post-intervention period stratified by perception of the leaflets in the practice (Leaflets = leaflets seen, No leaflets = leaflets not seen, don’t remember) (Leaflets for respiratory tract infections, Switzerland, 2022-2023)


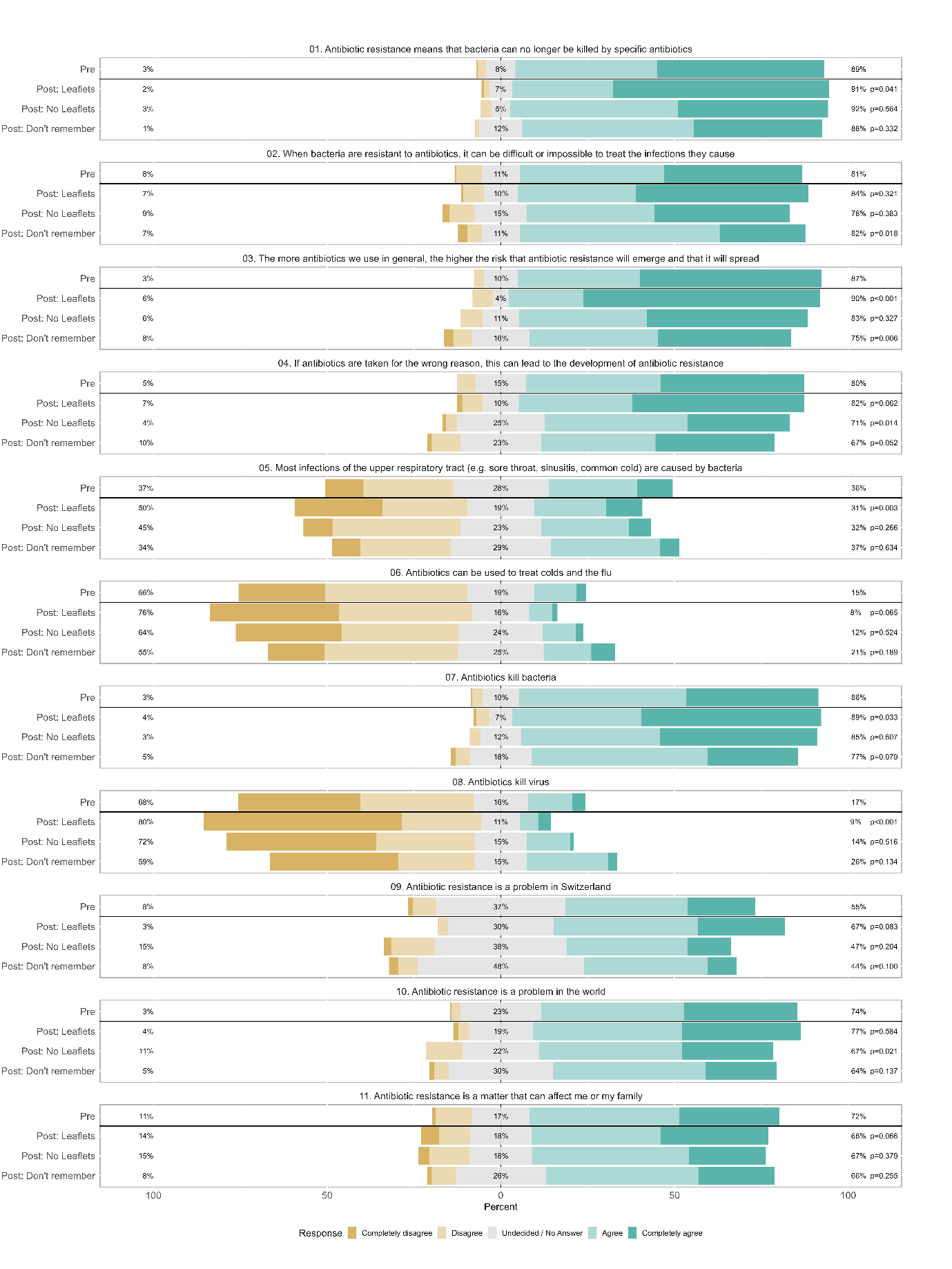


| **Checklist for Reporting Results of Internet E-Surveys (CHERRIES)** (Leaflets for respiratory tract infections, Switzerland, 2022-2023) | | |
| --- | --- | --- |
| **Checklist item** | **Explanation** | **Location** |
| **Design** | | |
| **Survey design** | Open online survey. | Methods section |
| **Institutional Review Board (IRB) approval and informed consent process** | | |
| **IRB approval** | As an anonymous survey, it did not fall under the scope of the Federal Act on Research Involving Human Beings, and the local ethics committee of the Canton of Zurich waived approval. | Methods section |
| **Informed consent** | Participants had to accept an informed consent statement on the opening page. The statement provided information on the purpose of the study, contact data of the investigators, and disclosure of potential conflicts of interest. Participants agreed to analysis of their anonymous response data and to publication of results. | Methods section |
| **Data protection** | Only the investigators had login data (username and password) to the survey software. | Checklist |
| **Development and pre-testing** | | |
| **Development and testing** | The survey was developed and implemented by the investigators. | Methods section |
| **Recruitment process and description of the sample having access to the questionnaire** | | |
| **Open survey versus closed survey** | Open survey. | Methods section |
| **Contact mode** | Recruitment in GP practices by information letters | Methods section |
| **Advertising the survey** | None. | Checklist |
| **Survey administration** | | |
| **Web/E-mail** | Web-based survey. | Methods section |
| **Context** | The survey was constructed with the REDCap study software. Appearance of the web survey was neutral. | Checklist |
| **Mandatory/voluntary** | Participation was voluntary. | Methods section |
| **Incentives** | All participants had the opportunity to take part in a raffle (one of 5 tablet computers). | Checklist |
| **Time/Date** | 01.10.2022 – 31.03.2023 | Methods section |
| **Randomization of items or questionnaires** | There was no randomization of items or questionnaires. | Checklist |
| **Adaptive questioning** | Adaptive questioning was used where appropriate. For instance, questions about the perception of the leaflets in the waiting rooms. | Checklist |
| **Number of Items** | 41 (maximum) | Checklist |
| **Number of screens (pages)** | 19 (maximum) | Checklist |
| **Completeness check** | Access to the respective next page was granted only after completion of a questionnaire page. | Checklist |
| **Review step** | Respondents could switch between pages and review answers using buttons for forward and backward navigation between subsequent pages. | Checklist |
| **Response rates** | | |
| **Unique site visitor** | Only participants or visitors providing an answer to the informed consent on the opening page of the survey could be identified as respondents. Calculation of view and participation rate was therefore not possible. | Checklist |
| **Completion rate** | Pre intervention period: 93%  Post intervention period: 95% | Checklist |
| **Preventing multiple entries from the same individual** | | |
| **Cookies used** | No. | Checklist |
| **Registration** | No. | Checklist |
| **Analysis** | | |
| **Handling of incomplete questionnaires** | Missing answers were reported descriptively. | Methods section |
| **Questionnaires submitted with an atypical timestamp** | Timestamps were not assessed. | Checklist |
| **Statistical correction** | No statistical correction was used. | Checklist |

| Patient survey: English translation | |
| --- | --- |
| Conditions of participation  The study was initiated by the Institute of Primary Care of the University of Zurich and has been reviewed by the Cantonal Ethics Committee Zurich (Req-2022-00369). This study is anonymous and your individual identify will not be known at any time. The data will be scientifically analyzed locally by the study team and will not be passed on or marketed at any time. According to current regulations, the data will be stored for 10 years. There is no influence or funding from the pharmaceutical industry. There are no conflicts of interest for any of the persons involved. The results of the study will be published (again, full anonymity) and will be used to improve medical care in Switzerland. If you have any questions about the study, please contact the Institute of Primary Care at any time (contact person: Dr. A. Plate. E-mail: Andreas.Plate@usz.ch).  There will be no compensation for participation in the study. All participants who have completely filled out the questionnaire have the option to participate in a raffle for 5 iPads. An email address is required for participation. Your email is used exclusively to contact the winners and is not linked to the response data.  This study will run for 6 months (October 2022 to March 2023). It is possible to participate more than once in this survey. |  |
| I agree with the terms and wish to compete the survey | 1: Yes  2: No |
| Basic survey information | |
| Please enter you participation code. You find the code on the front page of your information letter. | Free text |
| Have you already filled out the questionnaire in the past 6 months? | 1: Yes  2: No |
| Please transfer your diagnostic code from your information sheet. Your family doctor has marked your diagnosis on the information sheet.  The code is located on the bottom part of your information sheet. | A1: Rhinitis/Rhinosinusitis/Sinusitis  A2: Pharyngitis/Tonsillitis  A3: Bronchitis  B1: Streptococcal-Pharyngitis  B2: Influenza  B3: Covid-19 |
| Basic information regarding the consultation | |
| In which month was your doctor's visit when you received the invitation for this survey? | 1: October 2022  2: November 2022  3: December 2022  4: January 2023  5: February 2023  6: March 2023 |
| How many days have passed between your visit to the doctor and the completion of this survey | 1: The consultation was today  2: Between 1 and 5 days  3: more than 5 days |
| How many days have you had symptoms before the medical consultation? | Free text |
| Did you receive an antibiotic prescription in the last consultation where you received the information letter for this survey? | 1: Yes  2: No  3: Don’t know |
| Can you name the prescribed antibiotic? | Free text |
| Please estimate: How much total time did you spend in the waiting room (minutes)? | 1: <15 minutes  2: between 15 and 30 minutes  3: >30 minutes  4: don’t remember |
| Knowledge / awareness of antibiotic prescribing inappropriateness | |
| How much do you agree with these statements? |  |
| Antibiotic resistance means that bacteria would not be killed by specific antibiotics | 1: Strongly agree  2: Agree  3: Undecided  4: Disagree  5: Strongly disagree  6: No answer |
| If bacteria are resistant to antibiotics, it can be difficult or impossible to treat the infections they cause |  |
| The more antibiotics we use in general, the higher the risk that antibiotic resistance will emerge and that it will spread |  |
| When antibiotics are taken for the wrong reason, this can lead to the development of antibiotic resistance |  |
| Most infections of the upper respiratory tract (e.g. sore throats, sinusitis, common cold) are caused by bacteria |  |
| Antibiotics can be used to treat colds and the flu |  |
| Antibiotics kill bacteria |  |
| Antibiotics kill viruses |  |
| Antibiotic resistance is a problem in Switzerland |  |
| Antibiotic resistance is a problem in the world |  |
| Antibiotic resistance is a matter that can affect me or my family |  |
| Understanding, perceived relevance, implementation measures, and evaluation of the Smarter Medicine fact sheets. (post intervention period only) | |
| Did you observe information materials (like posters or flyers) on the topic of “antibiotic usage for respiratory tract infections” in you GP practice? | 1: Yes  2: No  3: don’t remember |
| Where did you notice the information materials? | 1: Waiting area – Posters  2: Waiting area – Flyer  3: Waiting area – electronic information on a screen.  4: Welcome area – Flyer  5: Doctors room – Flyer  6: Other location: Please give details.  7: no answer |
| Did the staff at your doctor's office specifically refer to the information materials? | 1: Yes – my doctor used the information material during the consultation  2: Yes – my doctor refers to the information material available in the practice rooms (for example in the waiting room)  3: Yes – the practice staff told me about the information materials  4: Other  5: No |
| I think the information materials were important for me | 1: Strongly agree  2: Agree  3: Undecided  4: Disagree  5: Strongly disagree  6: No answer |
| The information material enabled discussion with the GP about therapy possibilities for my infection |  |
| The information materials influenced the choice of an antibiotic prescription. |  |
| Overall, the information material was useful |  |
| Do you have recommendations to improve the information materials? | Free text |
| SDM-Q-9 | |
| Nine statements related to the decision-making in your consultation are listed below. For each statement, please indicate how much you agree or disagree. |  |
| My doctor made clear that a decision needs to be made. | 0: Completely disagree  1: Strongly disagree  2: Somewhat disagree  3: Somewhat agree  4: Strongly agree  5: Completely agree |
| My doctor wanted to know exactly how I want to be involved in making the decision. |  |
| My doctor told me that there are different options for treating my medical condition. |  |
| My doctor precisely explained the advantages and disadvantages of the treatment options. |  |
| My doctor helped me understand all the information |  |
| My doctor asked me which treatment option I prefer. |  |
| My doctor and I thoroughly weighed the different treatment options. |  |
| My doctor and I selected a treatment option together. |  |
| My doctor and I reached an agreement on how to proceed. |  |
| Basic patient characteristics | |
| How old are you? | Free text |
| What is your gender | 1: Male  2: Female  3: Other  4: I don’t want to answer |
| Are you a current smoker? | 1: Yes – active smoker  2: No - Never smoker  3: No - Former smoker |
| Do you have any of the following chronic diseases? | 1: Chronic lung disease  2: Chronic heart disease  3: Diabetes mellitus  4: Active Cancer  5: Chronic kidney disease  2: No  3: No answer |
| Raffle | |
| Thank you for participating in the survey. As a small thank you for taking part in this survey, you now have the opportunity to take part in a prize draw for a total of 4 iPads. For this purpose, please enter your raffle code and email address. You can find the raffle code on your information sheet.  The iPads will be drawn among all participants who have completed the questionnaire.  Participation in the raffle is voluntary. |  |
| Please enter your raffle code | Free text |
| Please enter your email address here | Free text |

Patient information leaflets

Available from: [Smarter Medicine Switzerland](https://www.smartermedicine.ch/de/angebot/infografiken-fuer-behandelnde) (link accessed August 2023)
